# Artificial Intelligence Techniques for Prostate Cancer Detection through Dual-Channel Tissue Feature Engineering

**DOI:** 10.3390/cancers13071524

**Published:** 2021-03-26

**Authors:** Cho-Hee Kim, Subrata Bhattacharjee, Deekshitha Prakash, Suki Kang, Nam-Hoon Cho, Hee-Cheol Kim, Heung-Kook Choi

**Affiliations:** 1 Department of Digital Anti-Aging Healthcare, Inje University, Gimhae 50834, Korea; chgmlrla0917@naver.com (C.-H.K.); heeki@inje.ac.kr (H.-C.K.); 2 Department of Computer Engineering, u-AHRC, Inje University, Gimhae 50834, Korea; subrata_bhattacharjee@outlook.com (S.B.); deeskhithadp96@gmail.com (D.P.); 3 Department of Pathology, Yonsei University Hospital, Seoul 03722, Korea; hee74@yuhs.ac

**Keywords:** artificial intelligence, tissue feature engineering, dual-channel, prostate cancer, texture analysis, binary classification, prostate cancer detection

## Abstract

**Simple Summary:**

Artificial intelligence techniques were used for the detection of prostate cancer through tissue feature engineering. A radiomic method was used to extract the important features or information from histopathology tissue images to perform binary classification (i.e., benign vs. malignant). This method can identify a histological pattern that is invisible to the human eye, which helps researchers to predict and detect prostate cancer. We used different performance metrics to evaluate the results of the classification. In the future, it is expected that a method like radiomic will provide a consistent contribution to analyze histopathology tissue images and differentiate between cancerous and noncancerous tumors.

**Abstract:**

The optimal diagnostic and treatment strategies for prostate cancer (PCa) are constantly changing. Given the importance of accurate diagnosis, texture analysis of stained prostate tissues is important for automatic PCa detection. We used artificial intelligence (AI) techniques to classify dual-channel tissue features extracted from Hematoxylin and Eosin (H&E) tissue images, respectively. Tissue feature engineering was performed to extract first-order statistic (FOS)-based textural features from each stained channel, and cancer classification between benign and malignant was carried out based on important features. Recursive feature elimination (RFE) and one-way analysis of variance (ANOVA) methods were used to identify significant features, which provided the best five features out of the extracted six features. The AI techniques used in this study for binary classification (benign vs. malignant and low-grade vs. high-grade) were support vector machine (SVM), logistic regression (LR), bagging tree, boosting tree, and dual-channel bidirectional long short-term memory (DC-BiLSTM) network. Further, a comparative analysis was carried out between the AI algorithms. Two different datasets were used for PCa classification. Out of these, the first dataset (private) was used for training and testing the AI models and the second dataset (public) was used only for testing to evaluate model performance. The automatic AI classification system performed well and showed satisfactory results according to the hypothesis of this study.

## 1. Introduction

In 2011, the World Health Organization (WHO) reported that cancer caused more deaths than strokes and coronary heart disease combined, and global demographics and epidemiological indications suggested that the trend would continue, especially in low-income countries. Annual cancer cases may exceed 20 million as early as 2025. In 2012, 14.1 million new cancer patients and 8.2 million cancer deaths occurred worldwide; lung, breast, colon, prostate, stomach and liver cancers accounted for 55% of the global burden. Prostate cancer (PCa) is common (1.1 million cases and 307,000 deaths annually) and confined to males [1,2]. Although optimal PCa diagnosis and treatment change constantly, the Gleason grading system is a good predictor.

**Figure 1:**
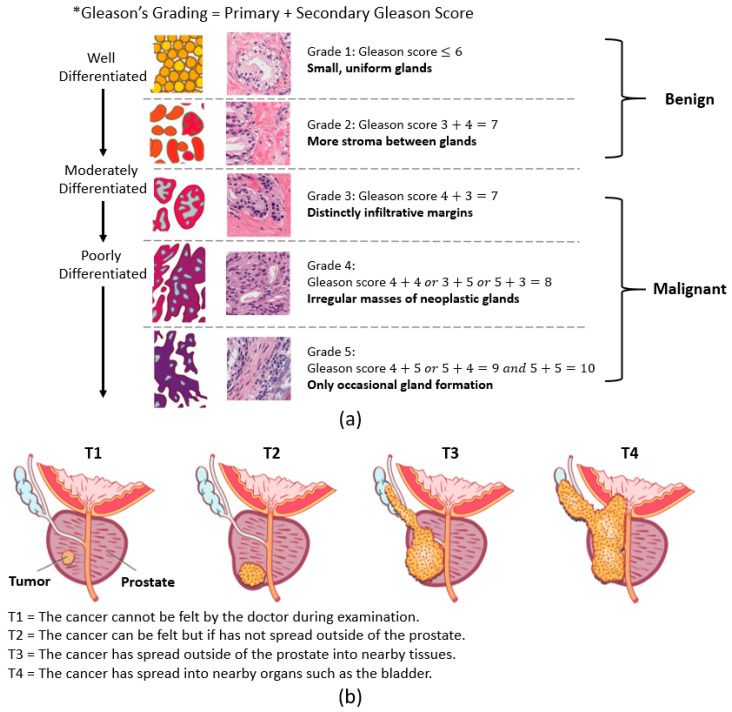
The grading and staging of prostate cancer. (**a**) The Gleason grading system. (**b**) The tumor staging system (Source: https://sunshinecoasturology.com.au/useful-info/urological-conditions/what-is-prostate-cancer/ accessed on 2 March 2021).

PCa stages are defined by the Gleason score and the tumor (T) score. The Gleason score, which ranges from 1 to 5, reflects tumor aggressiveness. The Gleason grading system is used if a biopsy finds cancer, which recognizes five stages of PCa according to gland shape and extent of differentiation [3,4]. Pathologists grade PCa based on the primary and secondary Gleason scores. The primary and secondary scores reflect the three factors used to grade cancer. On the other hand, the stages of PCa using the T score are measured by rating the size and extent of the original tumor. Here, the PCa is grouped into four stages (i.e., T1 to T4). Figure 1a,b shows the grading and staging of PCa.

Over the past 150 years, pathologists have microscopically evaluated tissue slides when evaluating cancer status, but this is difficult because only a minuscule proportion of observed cells may be tumorous. Improvements in diagnosis and more targeted treatments are needed [5,6]. An AI system may be helpful. Recently, DeepMind (Google) has dramatically reduced breast cancer diagnosis error [7]. Novartis is working with PathAI to develop AI for cancer diagnosis and treatment decision-making [8].

To perform cancer analysis and classification, machine learning (ML) is an appropriate method compared to visual analysis. The main difference between deep learning (DL) and ML classification is the way data is presented to the system. ML algorithms require organized data, while DL relies on layers of artificial neural networks (ANN). Generally, DL requires a large amount of data for better prediction and more computational time, whereas ML can predict accurately even with a small dataset and requires less computational time. Our study focuses on a texture feature-based classification using AI techniques. There are many tools provided by ML by which data can be analyzed and classified automatically.

Tissue images contain a lot of information (i.e., grey level patterns), which are difficult to analyze by eye because of the invisibility [9]. This textural information can be extracted and analyzed using a radiomics technique [10] and through the process of tissue feature engineering (i.e., the computation of tissue-level features). Feature selection optimally reduces computational complexity and improves classification accuracy and model performance. Features are the 2D spatial values of an image that represents the type of texture. In this paper, the recursive feature elimination (RFE) method was used to select the best features for AI classification. This method is popular and easy to configure because it is highly effective at selecting significant features in the dataset. Further, an ANOVA test was performed to identify the significant difference between benign and malignant features.

The texture is a key element of human-recognizable visual perception and is used in various ways in computer vision systems. It is easy for the human eye to distinguish different textures, but this can be perceived as a rather tricky problem on a computer. Among the techniques for analyzing textures, a structural approach is used for the statistical characteristics of images because the pattern of textures creates the structure, and the texture has different consistent properties. Statistical analysis methods indirectly express textures by nondecisive attributes that control the distribution and relationship between the intensity levels of the image. Accurate diagnoses and cancer grading are essential. Grade-specific features must be defined. Here, we use a feature extraction process called feature engineering to extract textural aspects from tissue images and differentiate malignant (grade 3, 4, and 5) from benign (grade 1 and 2) and grade 5 from grade 3 prostatic samples using the proposed AI models, which include support vector machine (SVM), logistic regression (LR), boosting tree, bagging tree, and dual-channel bidirectional long short-term memory (DC-BiLSTM). Pixel distribution analysis is very important to understand the variation of intensity in the image.

## 2. Related Work

Existing research on tissue texture analysis and classification has shown promising performance for PCa detection using Hematoxylin and Eosin (H&E) histopathology images. Most of the existing research focuses on binary classification for differentiating malignant and benign tumors using computer-aided diagnosis (CAD) tools. In the past, many researchers used an AI system to detect malignant biopsies and decrease the workload of pathologists. Therefore, the pathologist can be assisted through an AI system with the detection of PCa among the biopsies that are included in the preliminary screening process. In this section, we mainly discuss the methods of classification and feature extraction. Past studies related to AI-based classification are summarized in Table 1.

The studies in Table 1 show that different AI techniques and parameters have been used for cancer classification. Most of the studies performed binary classification using second-order statistical features to discriminate between noncancerous and cancerous tumors. Among them, Chakraborty et al. [21] achieved the best result in classifying the histopathologic scans of the lymph node section using a dual-channel residual convolution neural network. Similarly, the present study achieved astounding results in classifying the first-order statistic (FOS)-based texture features extracted from H&E channels, and considering all these different approaches, a comparative analysis was performed using the various AI models, namely SVM, LR, bagging tree, boosting tree, and DC-BiLSTM.

## 3. Data Collection and Image Representation

### 3.1. Data Collection

The following two datasets were collected from two different centers. Out of these, one was private and the other one was public. Both the datasets were used for preprocessing, prior to feature extraction and classification.

**Figure 2:**
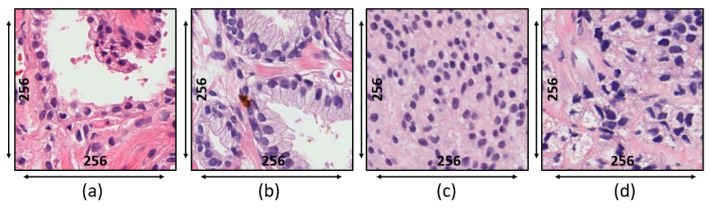
Microscopic biopsy images of a private dataset at the magnification factor of 40×. (**a**) Benign tumor. (**b**) Grade 3 tumor. (**c**) Grade 4 tumor. (**d**) Grade 5 tumor.

Private Dataset: The tissue slides used for this research were acquired from 20 patients and prepared at the Severance Hospital of Yonsei University, Korea. To prepare the tissue slides, the pathologist used the H&E staining system. Deparaffinization and rehydration were performed before H&E staining, as incomplete removal of paraffin wax compromises staining. Tissues were sectioned to 4 µm and autostained. Slides were scanned at 40× magnification using a 0.3-NA objective (Olympus BX-51 microscope) and photographed (Olympus C-3000 digital camera). Each slide contained 33,584 × 70,352 pixels. H&E-stained regions of interest (ROIs) of 256 × 256 pixels were extracted from whole slide images (WSIs), as shown in Figure 2. As shown in Table 2, 500 images were used for textural analysis, feature extraction, and classification, of which 250 were benign and 250 malignant (50 of grade 3, 100 of grade 4, and 100 of grade 5).

**Figure 3:**
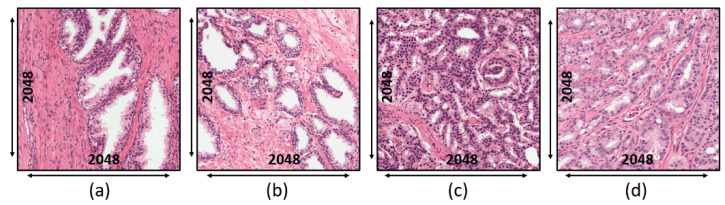
Microscopic biopsy images of an external test set at the magnification factor of 10×. (**a**,**b**) Benign tumor. (**c**,**d**) Malignant tumor.

External Test Set: The dataset was collected online and publicly available at https://zenodo.org/record/1485967#.X_0ue-gzZMs (accessed on 25 January 2021). Bulten et al. [22] made their dataset and uploaded it to the Zenodo repository, which can be used for external validation. A total of 102 patients experienced a radical prostatectomy at the Medical Center of Radboud University. Out of these, the H&E-stained samples of 40 patients were selected to check the performance of the models, of which the WSI for each patient was divided into four sections (i.e., two containing benign epithelium and two containing tumor). From each section, the ROIs of 2500 × 2500 pixels were extracted at 10× magnification, shown in Figure 3. As a result, 160 ROIs were extracted (89 of benign epithelial and 71 of tumor). The best 10 ROIs of benign epithelial and tumor were selected for model validation.

### 3.2. Image Representation

**Figure 4:**
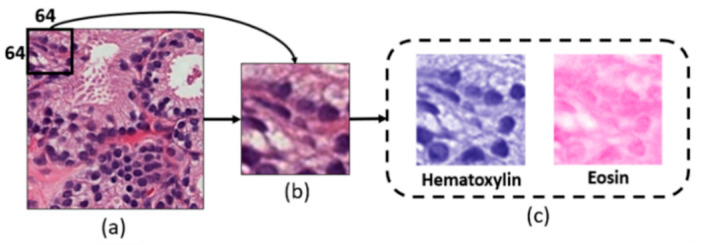
Block diagram of patch extraction and stain deconvolution of histopathological images. (**a**) The preprocessed regions of interest (ROI) after gamma correction. (**b**) Patch extraction of size 64 × 64 pixels (24-bits/pixel). (**c**) Hematoxylin and Eosin channels extracted from (**b**). The black bounding box in (**a**) represents the size of the sliding window for extracting the patches.

A power-law transformation (gamma correction) [23,24] was applied to the private dataset to adjust the contrast level of the tissue image. This method controls the overall brightness of an image and therefore, helps to display it accurately on a computer screen. Further, the preprocessed ROIs of H&E tissue samples (i.e., private dataset and external test set) were used for generating the non-overlapping patches of size 64 × 64 pixels, and a stain deconvolution technique was used to separate the Hematoxylin and Eosin channels from the extracted patches, as shown in Figure 4. A total of 8000 patches were selected from each dataset (private and external). Before generating patches of the external test set, the ROIs were resized to 2048 × 2048 pixels. According to the rule of thumb, the greater the learning samples per class [25], the better the model classification. Patch-based texture analysis was performed to increase the number of samples in the dataset and to extract more discriminating features.

## 4. Materials and Methods

**Figure 5:**
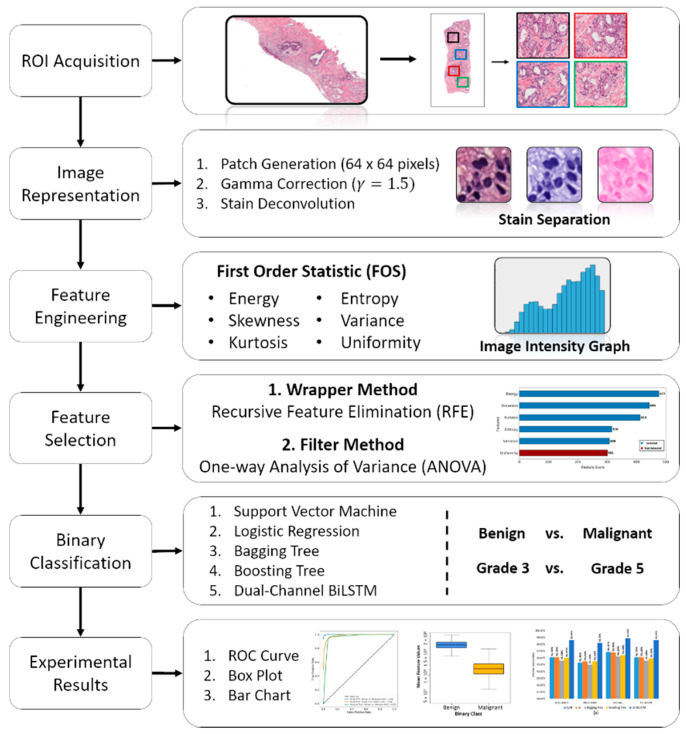
The proposed pipeline of the research work. Each step in the process flow diagram is carried out separately and independently.

The proposed pipeline of this study is depicted in Figure 5. First, after ROIs (256 × 256 pixels) acquisition from the stained WSI, the images were separated between two groups (benign and malignant). Second, the extracted ROIs were used for patch generation, gamma correction, and stain deconvolution. Third, for texture analysis, a set of radiomic features were extracted from dual-channel (Hematoxylin and Eosin) separately. Forth, two steps of feature selection (RFE and one-way ANOVA) were carried out to validate feature significance. Fifth, a binary classification was performed using the AI models. Finally, a comparative analysis of classification algorithms and performance evaluation was performed using a confusion matrix and receiver operating characteristic (ROC) curve.

### 4.1. Patch-Based Feature Engineering

The textural analysis exploits spatial changes in image patterns to extract information from both images and shapes. Such features are effective classifiers because they contain statistical data on adjacent pixels in images [26,27]. Here, we extracted image textural features and classified them using AI-based models. Radiomic is a technique of extracting a large number of features from captured visual content of medical images for analysis and classification. In this paper, we extracted FOS-based features in which the texture values were statistically computed from an individual pixel without considering the relationships of neighboring pixels [28]. The given radiomic features [29] that were extracted from H&E staining channels are given in the Supplementary Information (SI), Feature Extraction, and Table S1.

### 4.2. Features Selection

To select significant features from those extracted, we used two-step feature selection methods, namely wrapper (RFE) [30] and filter (one-way ANOVA) [31]. At times, due to insignificant input features, the learning algorithms could be deceived, resulting in poor predictive performance. Therefore, feature selection is an important step for AI-based classification, which selects the most relevant features for a dataset.

**Figure 6:**
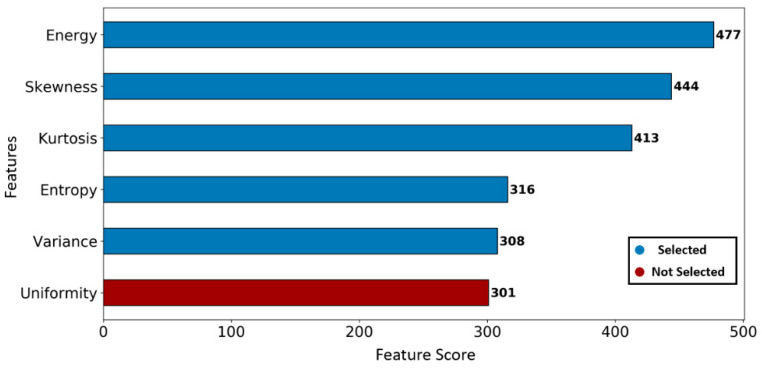
Feature ranking using the recursive feature elimination technique. The best five significant features are selected with the largest ranking values.

First, the best five features were selected using an RFE (greedy optimization algorithm) technique, which generates baseline models repeatedly and selects the strongest or weakest performing feature at each iteration until all the features are classified. In our study, a gradient boosting classifier was used as a baseline model to carry out the RFE process [32]. As a result, features were ranked based on the descending order from strongest to weakest, as shown in Figure 6.

Second, as shown in Table 3, a one-way ANOVA statistical test was performed to identify the feature significance (*F*-value and *p*-value) and effect size (i.e., eta squared) from those selected using RFE. The magnitude differences between the two groups (i.e., benign and malignant) were analyzed based on the eta squared and effective size (i.e., 0.01 = small, 0.06 = medium, and 0.14 = large) [33,34]. The small, medium and large effect sizes signify the difference between the two groups as unimportant, less important, and important, respectively. The eta squared is calculated using the following equation,

(1) $$\eta^{2}=\frac{{\mathrm{SS}}_{\mathrm{effect}}}{{\mathrm{SS}}_{\mathrm{total}}}$$

where ${\mathrm{SS}}_{\mathrm{effect}}$ is the sum of squares between the groups, ${\mathrm{SS}}_{\mathrm{total}}$ is the sum of squares between + within the groups, and $\eta^{2}$ is the eta squared.

### 4.3. Binary Classification

The classification of textural features from medical images provides useful information and helps pathologists to make accurate decisions. In this paper, we used AI-based algorithms to perform binary classification and differentiate malignant from benign tissue and high-grade (grade 5) from low-grade (grade 3) tissue samples. ML and DL-based classification are very important to understand the image patterns of a certain disease. To perform the ML classification, extracted features from H&E are concatenated before initiating the learning process. In contrast, for DL-based DC-BiLSTM classification, the dual-channel features from H&E are concatenated through the learning process. In this work, multiple ML and DL-based algorithms were developed, and we carried out a comparative analysis to determine performance based on the evaluation metrics. A detailed explanation of the classification methods is given in the Supplementary Information (SI) and Classification Algorithms [35,36,37,38,39,40,41,42,43,44,45]. In the Supplementary Information, Figures S1–S3 show the classification process of Bagging and Boosting and LSTM algorithms, respectively.

**Figure 7:**
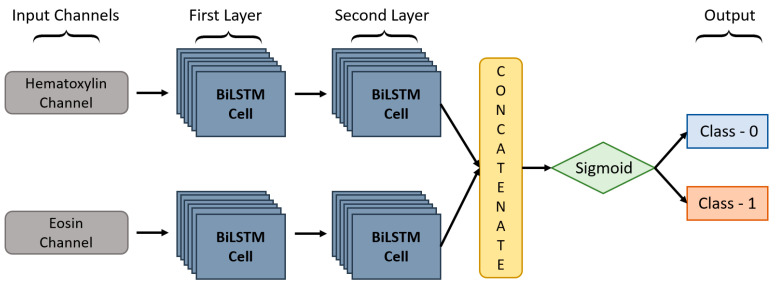
The structure of the proposed DC-BiLSTM Network.

In this paper, we proposed a DC-BiLSTM model for learning dual-channel tissue features and discriminating diseases based on normal and abnormal prostate tissues, which is a novel approach. In general, a unidirectional LSTM model consists of one LSTM that works only in one way, learning the input features from the past to the future in a forward direction. On the other hand, a BiLSTM [46] model consists of two LSTMs that work in two ways, one learning the inputs from past to future and the other from future to past in a forward and backward direction. In BiLSTM, the information learned from both directions is concatenated for the final computation. As shown in Figure 7, DC-BiLSTM network consisted of two input channels for learning Hematoxylin and Eosin-based tissue features. Two layers of BiLSTM were used for each input channel containing the same number of nodes (i.e., time steps and cells = 64). The model concatenated the outputs of the two channels into a single feature vector and passed them to a fully connected layer for cancer classification.

The hypothesis we created for the binary classification is as follows:

(a) For the internal test set, the recall of benign vs. malignant and grade 3 vs. grade 5 classification must be ≥90% and ≥80%, respectively.
(b) For the external test set, the recall of benign vs. malignant classification must be ≥85%.

## 5. Experimental Results

### 5.1. Model Performance

The implementation (i.e., image representation, feature extraction, feature selection, model classification) for this study was carried out using MATLAB and Python programming on an Intel Core i7 workstation with 24 GB RAM. For the analysis and classification of dual-channel tissue features, we used both private and public datasets. For the private dataset of benign and malignant tissue, we extracted 8000 patches from 500 ROIs of size 256 × 256 pixels. Out of these, 5120 were for training, 1280 for validation, and 1600 for testing. Moreover, within the malignant tissues, 1600 patches were extracted separately from 100 ROIs (50 of grade 3 and 50 of grade 5) to validate the performance of the trained classifiers in distinguishing between low-grade and high-grade disease. On the other hand, for the external test set, we extracted 10,240 patches from 10 ROIs of size 2048 × 2048 pixels, of which the best 8000 patches, excluding background, were selected for model validation in distinguishing between benign and malignant tissues. The evaluation metrics used to compute the results of binary classification were accuracy, recall, precision, and f1-score. Table 4, Table 5 and Table 6 show the test results and comparative analysis of the multiple learning algorithms for the private and public datasets. For the internal test sets of benign vs. malignant and grade 3 vs. grade 5, the best classification results obtained by DC-BiLSTM were accuracy = 98.6% and 93.5%, precision = 98.2% and 96.3%, recall = 98.9% and 91.2%, and F1 score = 98.6% and 93.7%, respectively. For the external test set of benign vs. malignant, the best classification results obtained by Boosting Tree were accuracy = 93.5%, precision = 92.9%, recall = 94.1%, and F1 score = 93.5%.

### 5.2. Result Evaluation

It was found from the comparative analysis that DC-BiLSTM and Boosting tree outperformed all the other classifiers. In general, for evaluating the performance of the classification model, the N × N confusion matrix is used which compares the target labels with those predicted by the models. Therefore, we used 2 × 2 matrices for evaluating the results of binary classification. Table 7, Table 8 and Table 9 show confusion matrices for the internal and external test sets with magnification factors of 40× and 10×, respectively. In classifying benign and malignant tissue, the confusion matrix of each classifier demonstrated that the malignant samples were more accurately classified compared to benign samples. This is because the tissue texture between malignant and benign samples was very different from each other. However, in the malignant dataset, low-grade samples were also included whose texture pattern was fairly similar to benign. Consequently, the classifier identified some of the low-grade samples as benign and the misclassification rate of benign increased gradually.

**Figure 8:**
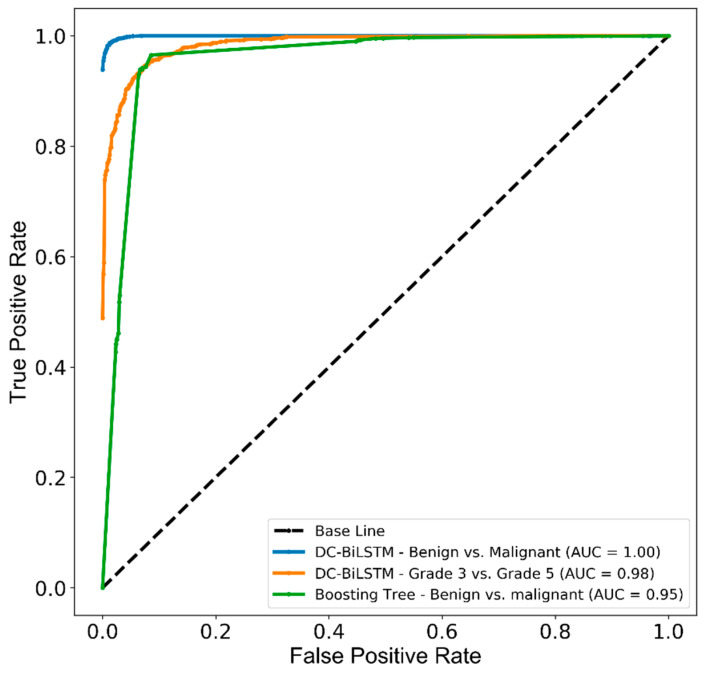
The receiver operating characteristic (ROC) and area under the curve (AUC) for PCa classification between benign and malignant tissues, and within malignant tissue samples. The test performance of DC-BiLSTM and Boosting tree classifiers used the internal and external test datasets.

Figure 8 shows the receiver operating characteristic (ROC) curve and area under the ROC curve (AUC), which was generated to measure and compare the usefulness of the optimum AI models. AUCs of 1.00, 0.98, and 0.95 were achieved by the AI system representing the ability to distinguish malignant from benign and grade 5 from grade 3 tissue samples.

## 6. Discussion

We developed an AI-based CAD system for PCa classification that can achieve outstanding discrimination between benign and malignant biopsy tissue samples. This system can classify only binary samples for cancer detection. The initial step of this work was to analyze the ROIs of benign, grade 3, grade 4 and grade 5 tissue samples. We used QuPath open-source software for analyzing the tissue samples manually and separated the ROIs into two classes, namely benign and malignant (i.e., grade 3, grade 4, and grade 5). In benign samples, the glands are small and uniform in shape, with more stroma between glands, whereas in malignant samples, there are irregular masses of neoplastic glands, absence of glands and sheets of cells. Tissue-level texture analysis was performed for differentiating malignant from benign tumors and grade 5 from grade 3 tumors. In general, we analyzed the image texture by calculating the magnitude of the pixel values, peaks of the distribution values, randomness in the image values, homogeneity of the image intensity values, the coarseness of image texture, gray level intensity and a grouping of pixels with similar values.

Before we performed feature extraction, selection, and classification, we generated small patches of size 64 × 64 pixels from the selected ROIs and used the stain deconvolution method to separate the H&E channels. FOS-based features (energy, entropy, kurtosis, skewness, variance and uniformity) were calculated from each staining channel separately. Out of six extracted features, the best five were selected using RFE and a one-way ANOVA technique based on the feature ranking, *p*-values and effect size (eta squared). Later, the significant features of the two channels (H&E) were concatenated and classified using the AI models. Although the external test set was unknown for the learning models, the AI system performed well in the binary classification. The bar charts of the comparative analysis are given in the Supplementary Information (SI) and Figures (Figure S4a–c).

**Figure 9:**
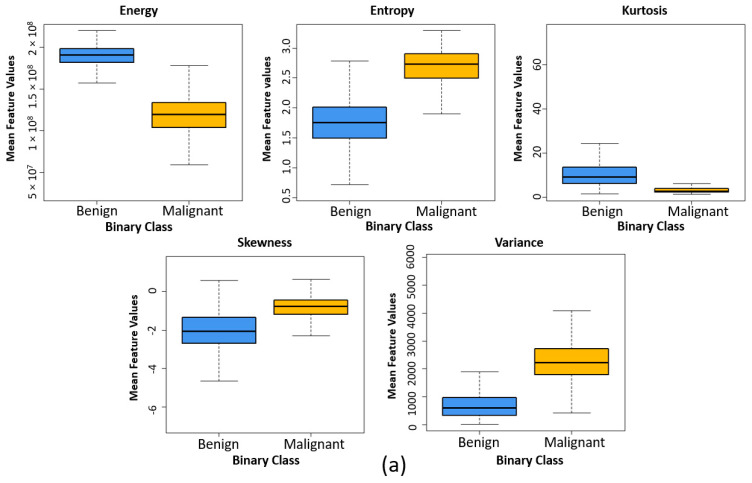

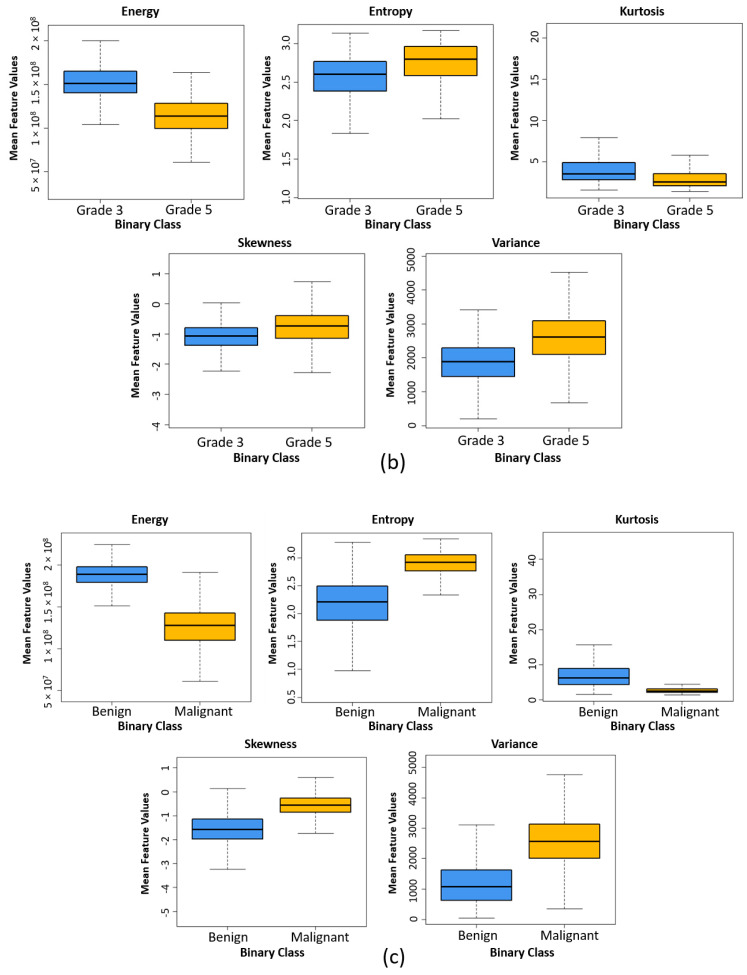
Comparing the texture differences between benign and malignant tissue and grade 3 and grade 5 tissue samples using five high-ranked radiomic features. (**a**) Box plot comparing the five high high-ranked radiomic features between benign and malignant (internal test set). (**b**) Box plot comparing the five high high-ranked radiomic features between grade 3 and grade 5 (internal test set). (**c**) Box plot comparing the five high high-ranked radiomic features between benign and malignant (external test set).

Figure 9 shows the box plots for two different groups used for binary classification. The box plots were generated by calculating the mean feature values of each group. Five high-ranked radiomic features extracted from independent and external test sets were used for comparing the texture differences between benign and malignant and grade 3 and grade 5 tissue samples. It can be observed that the structure of box plots and the mean values for each feature in Figure 9a,c are quite similar, which demonstrates that the texture of prostatic tissue of independent and external test sets was relatively comparable. As a result, the classification algorithms had a good chance in accurate classification and making the right decision.

In our previous study [47], we performed textural analysis and extracted a total of 12 radiomic features (second-order statistic) using the gray-level co-occurrence matrix (GLCM) method, of which the best 10 features were selected using one-way ANOVA. The image size was 512 × 512 pixels (24 bits/pixel). Feature classification was performed separately with 10 and 12 features using SVM and *K*-nearest neighbors (KNN) classification algorithms. The training-to-testing sample ratio was 8:2. The classification accuracies for SVM were 81.6% and 84.1% using 12 and 10 features, respectively, and the accuracies for KNN were 77.5% and 79.1% using 12 and 10 features, respectively. In the present study, we used the window size of 64 × 64 to extract 16 patches from a single image of size 256 × 256 pixels. The number of data samples for benign and malignant tissues were limited, and, therefore, patch extraction was performed to boost the training sample per class. The FOS features were extracted from H&E staining channels after image representation was performed and, therefore, our AI system uncovered effective results for the detection of PCa. Even though our proposed H&E network (DC-BiLSTM) achieved high accuracy using the internal test sets, it did not perform very well for the external test set compared to the Boosting tree classifier. This is because the proposed network was developed and fine-tuned based on the private dataset and, in the histological sections, the spatial distributions of an image differed from one dataset to another. It was difficult to determine the performance of the trained model using the external or blind test set. However, the proposed network performed well and achieved satisfactory results according to the hypothesis of this study.

In medical image processing, feature extraction and selection of key features is very important for microscopic biopsy, magnetic resonance (MR), ultrasound and x-ray image analyses. However, many researchers use doctors’ recommended clinical features for disease classification and, therefore, cannot achieve better results. The texture of medical images provides a lot of information in the spatial arrangement of colors or intensities. A radiomic method can be used to extract this information for AI-based disease classification, and thus better diagnostic results can be obtained.

## 7. Conclusions

We used various FOS features to perform AI-based classification and analyzed textural dissimilarities in prostate tissue images. The purpose of this paper was to analyze the significant features and classify them for PCa detection. Two-step feature selection was effective in terms of selecting important features. Our models yielded promising results using FOS radiomic features extracted from patch images. We evaluated the performance and strength of the models using private and public datasets collected from two different centers. All the AI models achieved high recall in classifying benign and malignant tissue samples, which is very helpful for researchers and clinicians. Each model was successfully validated using the two internal and one external test datasets, achieving accuracies of 96.1, 85.2 and 88.2% using SVM; 96.1, 85.1 and 87.9% using LR; 95.6, 80.8 and 91.3% using Bagging tree; 96.0, 86.0 and 93.5% using Boosting tree and 98.6, 93.6 and 89.2% using DC-BiLSTM, respectively.

In this study, fine-tuning of the classification models was performed to reduce the overfitting problem. The performance evaluation of the AI models was carried out using 2 × 2 confusion matrices and ROC curves. Texture analysis of patch-based histopathological images is sometimes difficult due to spatial changes in image patterns. Therefore, some preprocessing, like smoothing effect, image normalization and intensity correction, is necessary to overcome this type of difficulty. However, in this study, gamma correction and stain deconvolution technique techniques were incorporated to adjust the intensity level and separate the staining channels of the tissue images, respectively. To analyze the texture of tissue images and extract significant information, we must use feature-engineered radiomics techniques. In future studies, it is highly recommended that the validation of AI models should be performed using other histopathological datasets containing various cancer cases.

## Figures and Tables

**Table 1:** Past studies related to cancer detection using artificial intelligence (AI) techniques.

Author	AI Techniques	Classification Types	Parameters	Performance
Mohanty et al., 2011 [11]	Association Rule	Binary (benign vs. malignant)	GLCM and GLRLM features	The association rule method was used for image classification. Accuracies of 94.9% and 92.3% were achieved using all and significant features, respectively.
Neeta et al., 2015 [12]	SVM and K-NN	Binary (benign vs. malignant)	GLDM and Gabor features	Detection of PCa using CAD algorithm and the best result of 95.83% was achieved using an SVM classifier.
Filipczuk et al., 2012, [13]	K-NN	Binary (benign vs. malignant)	GLCM and GLRLM features	Breast cancer diagnosis was performed by classifying the texture features based on GLCM and GLRLM extracted from the segmented nuclei. The best result of 90% was obtained by combining the optimal features of GLRLM.
Radhakrishnan et al., 2012 [14]	SVM	Binary (benign vs. malignant)	Histogram, GLCM, and GLRLM features	TRUS medical images were used for prostate cancer classification. The DBSCAN clustering method was used for extracting the prostate region. The best accuracy of 91.7% was achieved by combining the three feature extraction methods.
Sinecen et al., 2007 [15]	MLP1, MLP2 RBF, and LVQ	Binary (benign vs. malignant)	Image texture based on Gauss-Markov random field, Fourier transform, stationary wavelets	Prostate tissue images of 80 benign and 80 malignant cell nuclei were evaluated. The best accuracy of 86.88% was achieved using MLP2.
Bhattacharjee et al., 2019 [16]	MLP	Binary (benign vs. malignant)	Color moment and GLCM features	Wavelet-based GLCM and color moment descriptor were extracted from prostate tissue images of benign and malignant classes. The model achieved an accuracy of 95%.
Song et al., 2018 [17]	DCNN	Binary (cancer vs. non-cancer)	MRI scans	PCa and noncancerous tissues were distinguished using DCNN. An AUC of 0.944, a sensitivity of 87.0%, a specificity of 90.6 PPV of 87.0%, and an NPV of 90.6% were achieved using DCNN.
Bhattacharjee et al., 2019 [18]	SVM	Binary (benign vs. malignant) and Multiclass (benign vs. grade 3 vs. grade 4 vs. grade 5)	Morphological features	Morphological feature classification was performed for discriminating benign from malignant tumor, grade 3 from grade 4, 5 tumors, and grade 4 from grade 5 tumor. The best rest was obtained from binary classification.
Zhao et al., 2015 [19]	ANN	Binary (PCa vs. non-Pca)	GLCM, Gray-level histogram, and general features	T2-weighted prostate MRI scans were used to extract 12 different types of features. Feature classification was performed using an artificial neural network. For PZ and CG, the accuracies achieved using a CAD system were 80.3% and 84.0%, respectively.
Roy et al., 2019 [20]	CNN	Binary (nonmalignant vs. malignant) and Multiclass (normal vs. benign vs. in situ vs. invasive carcinoma)	Histology images	The patch-based classifier using CNN was developed for the automated classification of histopathology images. In classifying the images of the cancer histology test dataset, the proposed technique achieved promising accuracies for both binary and multiclass classification.
Chakraborty et al., 2020 [21]	DCRCNN	Binary (cancer vs. noncancer)	Histopathologic scans	A dual-channel residual convolution neural network was used to classify the tissue images of the lymph node section. The model was trained with 220,025 images and achieved an overall accuracy of 96.47%.

**Table 2:** The numbers of benign and malignant images used for training and testing. Private dataset structure at 40× magnification factor.

Groups	Training		Testing	Total
	Train	Validation		
Benign	160	40	50	250
Malignant	160	40	50	250
Total	320	80	100	500

**Table 3:** The second step feature selection using one-way ANOVA. Significant features are identified based on *p*-value < 0.05, and large effect size.

Feature Name	Significance		Effect Size
	*F*-Value	*p*-Value	Eta Squared
Energy	25,550.5	<0.05	0.77127
Skewness	3375.6	<0.05	0.32936
Kurtosis	2351.6	<0.05	0.35199
Entropy	5742.8	<0.05	0.58838
Variance	5689.3	<0.05	0.58672

**Table 4:** Comparative analysis of the performance of multiple classifiers in distinguishing between benign and malignant tissue. The performance metrics are for the internal test dataset.

Models	Accuracy	Precision	Recall	F1-Score
SVM	96.1%	95.3%	96.9%	96.1%
LR	96.1%	95.5%	96.8%	96.1%
Bagging Tree	95.6%	95.0%	96.2%	95.6%
Boosting Tree	96.0%	95.5%	96.4%	95.9%
DC-BiLSTM	98.6%	98.2%	98.9%	98.6%

**Table 5:** Comparative analysis of the performance of multiple classifiers in distinguishing between low-grade and high-grade disease. The performance metrics are for the internal test dataset.

Models	Accuracy	Precision	Recall	F1-Score
SVM	85.2%	89.5%	82.4%	85.8%
LR	85.1%	89.5%	89.2%	85.7%
Bagging Tree	80.8%	80.8%	80.8%	80.8%
Boosting Tree	86.0%	84.7%	86.9%	85.8%
DC-BiLSTM	93.6%	96.3%	91.2%	93.7%

**Table 6:** Comparative analysis of the performance of multiple classifiers in distinguishing between benign and malignant tissue. The performance metrics are for the external test dataset.

Models	Accuracy	Precision	Recall	F1-Score
SVM	88.2%	83.1%	92.6%	87.6%
LR	87.9%	82.2%	92.1%	87.3%
Bagging Tree	91.3%	87.5%	94.7%	91.0%
Boosting Tree	93.5%	92.9%	94.1%	93.5%
DC-BiLSTM	89.2%	88.7%	90.0%	89.2%

**Table 7:** Confusion matrices of classification algorithms—Malignant vs. Benign (internal test set).

**Binary Classification**	**SVM**			**LR**		**Bagging Tree**		
	**Benign**	**Malignant**		**Benign**	**Malignant**	**Benign**		**Malignant**
Benign	763	37		764	36	760		40
Malignant	24	776		25	775	30		770
	**Boosting Tree**				**DC-BiLSTM**			
	**Benign**		**Malignant**		**Benign**		**Malignant**	
Benign	764		36		786		14	
Malignant	28		772		8		792	

**Table 8:** Confusion matrices of classification algorithms—Grade 3 vs. Grade 5 (internal test set).

**Binary Classification**	**SVM**			**LR**		**Bagging Tree**		
	**Grade 3**	**Grade 5**		**Grade 3**	**Grade 5**	**Grade 3**		**Grade 5**
Grade 3	716	84		716	84	647		153
Grade 5	153	647		155	645	153		647
	**Boosting Tree**				**DC-BiLSTM**			
	**Grade 3**		**Grade 5**		**Grade 3**		**Grade 5**	
Grade 3	678		122		771		29	
Grade 5	102		698		74		726	

**Table 9:** Confusion matrices of classification algorithms—Malignant vs. Benign (external test set).

**Binary Classification**	**SVM**			**LR**		**Bagging Tree**		
	**Benign**	**Malignant**		**Benign**	**Malignant**	**Benign**		**Malignant**
Benign	3327	673		3318	682	3503		497
Malignant	266	3734		283	3717	195		3805
	**Boosting Tree**				**DC-BiLSTM**			
	**Benign**		**Malignant**		**Benign**		**Malignant**	
Benign	3719		281		3550		450	
Malignant	233		3767		409		3591	

## Data Availability

Private dataset (i.e., prostate tissue samples) is not available online. The data used in this study are available on request from the corresponding author. The public dataset presented in this study is openly available in the Zenodo repository at https://zenodo.org/record/1485967#.YEXth44zaUl (accessed on 20 January 2021). Code and test data is openly available in the Github repository at https://github.com/subrata001/Cancer-Classification-Through-Dual-Channel-Feature-Engineering (accessed on 8 March 2021).

## Supplementary Materials

The following are available online at https://www.mdpi.com/article/10.3390/cancers13071524/s1, Figure S1: An example of Bagging Tree classifier. Here, the classification is carried out in a parallel direction, Figure S2: An example of Boosting Tree classifier. Here, the classification is performed in a sequential direction, Figure S3: The operation and structure of LSTM cell. Figure S4: Comparative analysis graphs of four different evaluation metrics that show the results of binary classification obtained using different AI models. Table S1: The description and formula of the extracted FOS features.
