# Supplementary material for: Artificial Intelligence Techniques for Prostate Cancer Detection through Dual-Channel Tissue Feature Engineering
Source: Cancers (Basel). 2021 Mar 26;13(7):1524. doi: 10.3390/cancers13071524 (PMC8036750; doi:10.3390/cancers13071524)
Supplement: Supplementary file 1 [file cancers-13-01524-s001.pdf]

cancers MDPI

Supplementary Material: Artificial Intelligence Techniques for Prostate Cancer Detection through Dual-Channel Tissue Feature Engineering

Cho-Hee Kim, Subrata Bhattacharjee, Deekshitha Prakash, Suki Kang, Nam-Hoon Cho, Hee-Cheol Kim and Heung-Kook Choi

Supplementary Information (SI)

1. Feature Extraction

Table S1. The description and formula of the extracted FOS features.

Feature Name	Description	Formula
Energy	It computes the magnitude of pixel values in an image.	$\sum_{i=1}^{N_p}(\mathbf{X}(i)+c)^2$
Skewness	It calculates the asymmetry of the distribution of values about the mean value.	$\frac{\mu_3}{\sigma^3}=\frac{\frac{1}{N_p}\sum_{i=1}^{N_p}(\mathbf{X}(i)-\bar{X})^3}{\left(\sqrt{\frac{1}{N_p}\sum_{i=1}^{N_p}(\mathbf{X}(i)-\bar{X})^2}\right)^3}$
Kurtosis	It calculates the peakedness of the distribution of values in the image ROI.	$\frac{\mu_4}{\sigma^4}=\frac{\frac{1}{N_p}\sum_{i=1}^{N_p}(\mathbf{X}(i)-\bar{X})^4}{\left(\frac{1}{N_p}\sum_{i=1}^{N_p}(\mathbf{X}(i)-\bar{X})^2\right)^2}$
Entropy	It measures the randomness in the image values.	$-\sum_{i=1}^{N_g}p(i)log_2(p(i)+\epsilon)$
Variance (σ^2)	It measures the spread of distribution about the mean.	$\frac{1}{N_p}\sum_{i=1}^{N_p}(\mathbf{X}(i)-\bar{X})^2$
Uniformity	It measures the sum of squares of each intensity value and computes the homogeneity of the image intensity values.	$\sum_{i=1}^{N_g}\mathrm{p}(i)^2$

$\mathbf{X}$:	A set of ROI pixels (N_p)
$\bar{X}$:	Mean of the distribution
$\mathbf{P}$(i):	The first order histogram with the discrete intensity level N_g
N_g:	The number of non-zero bins of a histogram
p(i):	First-order normalized histogram, which is equal to $\frac{\mathbf{P(i)}}{N_p}$
ϵ:	An arbitrarily small positive number
μ_3 & μ_4:	The 3rd and 4th central moment
σ:	Standard deviation

2. Classification Algorithms

2.1. Support Vector Machine

The three important elements of an SVM are the margin, support vector, and kernel. The margin is the distance between the support vector and a hyperplane; when there are multiple hyperplanes, the margin finds the most reasonable hyperplane in the vector space. A support vector is the data vector closest to a hyperplane. The support vector and margin depend on the decision boundary (the hyperplane). Using the kernel, SVMs efficiently perform non-linear classifications by mapping inputs into high-dimensional feature spaces [35,36]. It is important to understand how decision boundaries are defined and calculated. We used a non-linear Gaussian kernel to find the points closest to the decision boundaries for both benign and malignant tumors. We used five-fold cross-validation to train our model; the training data were divided into five trials, and the validation accuracy was the average of the accuracies of the five trials. Cross-validation reduces over-fitting. Parameter tuning is critical; the C and γ parameter control SVM performance by setting the trade-off between the decision boundary and correct classification of the training data. A well-tuned C and γ are vital. The kernel function, used for binary classification can be expressed by:

$$\mathrm{D} = \sqrt{\left(\sum_{i=1}^{n}(x_i - y_i)^2\right)}$$

$$K(x_i, y_i) = \exp(-\gamma \times D^2), \gamma = \frac{1}{2\sigma^2} \quad (1)$$

where x_i, y_i is the feature vector in dimension n, D is the Euclidean distance between two feature vectors, γ is a hyper-parameter, which changes the smoothness of the kernel function, and σ is a free parameter.

2.2. Logistic Regression

LR is a linear algorithm that predicts the probability of the data that falls into a category between 0 and 1, and categorizes it as belonging to a higher probability category. In general, LR is applied for binary classification when the data samples are divided into two groups (e.g., positive and negative). We used this method in our paper to classify benign and malignant samples separately and independently. A linear return can easily predict unforeseen problems with logit transformation,

$$\mathrm{f(r)} = \frac{1}{1 + \mathrm{e}^{-\sum_{\mathrm{i=0}}^{\mathrm{n}} \mathrm{w_i x_i}}} \quad (2)$$

The classification is usually based on 0.5, a critical value. It can be changed to allow better classification. Here, e is the Eular′ number, w is coefficient of regression, x is the constant variable, and n is the number of features [37].

2.3. Bagging Tree

It is an abbreviation of Bootstrap Aggregation and is a classification method that makes the predictions by learning each model and aggregating the results. One of the biggest advantages of Bagging is that it can perform the parallel computation of the predictions. Generally, it occurs underfitting due to high bias and overfitting problems due to high dispersion, therefore ensemble voting technique can minimize these errors. In general, Bagging can solve these problems because it predicts the outcome by voting/averaging the results from each learning algorithm [38,39]. Figure S1 shows the process and technique of the Bagging Tree classifier. The principle of the bagging tree can be expressed by the following equation:

$$f(x) = \frac{1}{T}\sum_{t=1}^{T} f_b(x) \quad (3)$$

where T is the number of classifier and f_b is the weak learner on the boostraped dataset.

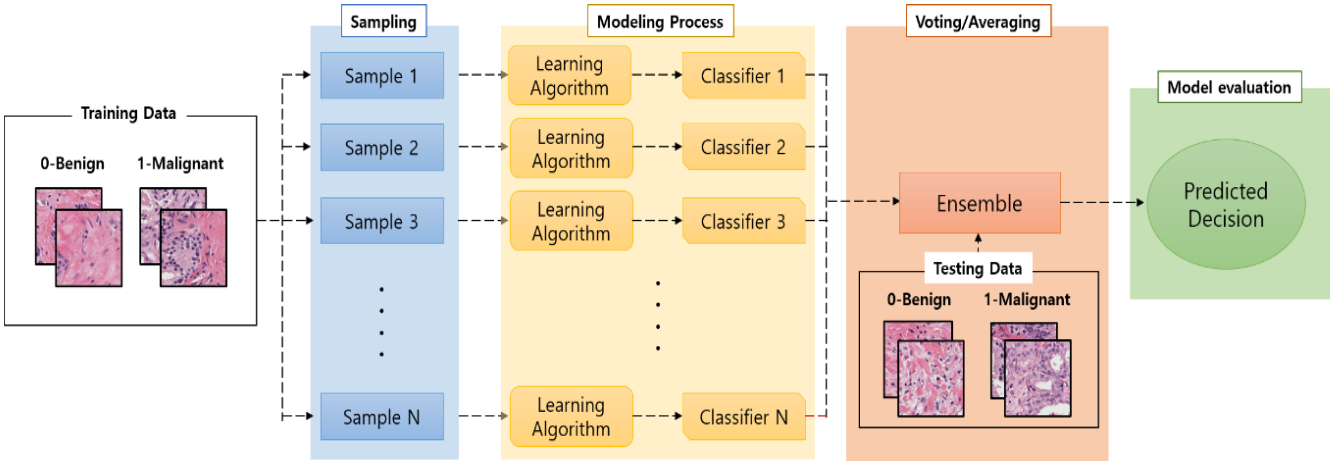

Figure S1. An example of Bagging Tree classifier. Here, the classification is carried out in a parallel direction.

2.4. Boosting Tree (AdaBoost)

Boosting Tree is commonly used in regression and classification as a general technique. It is one of the machine learning ensemble techniques that improve classification performance by making weak leaders into strong leaders. On the other hand, the Bagging tree is learned in a parallel track, while Boosting tree classification is performed sequentially. Once the learning is completed, the weights of the network are measured according to the results where the weights assigned affect the prediction outcome of the next predictive model [40]. Figure S2 shows the process and technique of the Boosting Tree classifier. The principle of the boosting tree can be expressed by the following equation:

$$f(x) = \sum_{t=1}^{T} \alpha_t h_t(x) \quad (4)$$

where T is the number of classifiers, α_t is the calculated weight, and $h_t(x)$ is the output of the weak classifier t.

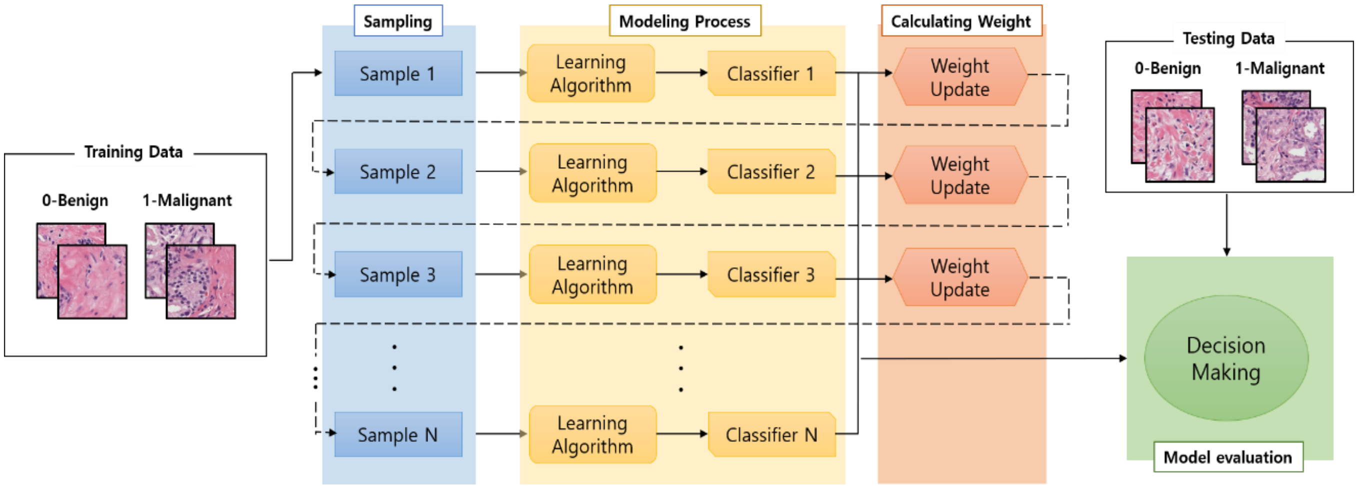

Figure S2. An example of Boosting Tree classifier. Here, the classification is performed in a sequential direction.

2.5. Dual-Channel BiLSTM

Long Short-Term Memory Network (LSTM) [41,42] is known as a groundbreaking model for the artificial recurrent neural network (RNN). It is used in the field of DL. RNN is an optimized model for dealing with sequential, listed data, and is used in various fields such as speech recognition, language modeling, etc [43, 44]. There are long-term dependency problems with RNN and therefore LSTM is proposed as a way to address these problems. LSTM consists of a memory cell, input gate, output gate, and forget gate, and the key idea is to open and close the gate for a long or short period. Figure S3 shows the structure of the LSTM cell and illustrates the basic operations of the gates [45].

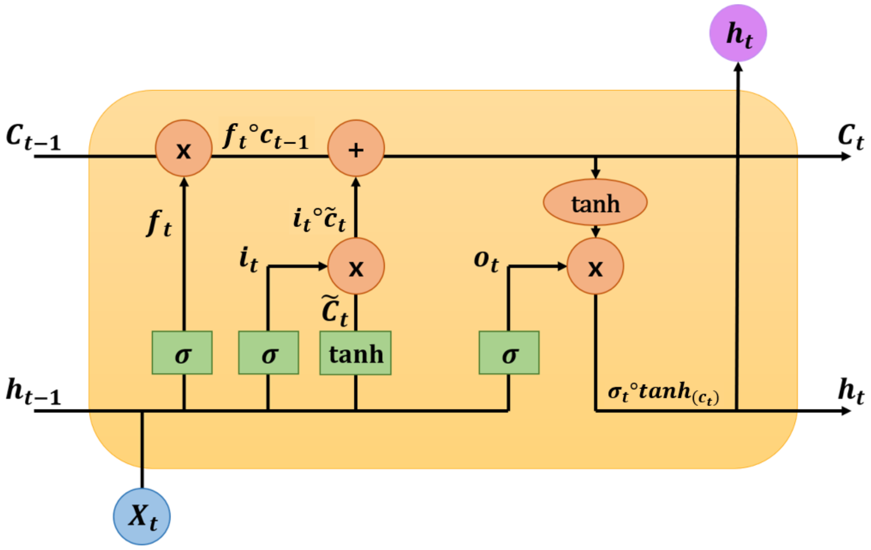

Figure S3. The operation and structure of LSTM cell. Reproduced from Olah [45].

The LSTM network has different steps to identify information during the learning process. The sigmoid function is used in the network as the gating function. In the first step, the sigmoid function is used in the forget gate layer to decide what information should be ignored from the cell state C_{t-1}. It takes the information from x_t at time t and h_{t-1} at time $t-1$ and outputs a value between 0 (i.e., remove information) and 1 (i.e., keep information). The equation for the forget gate can be expressed by:

$$f_t = \sigma(W_f \cdot [h_{t-1}, x_t] + b_f) \tag{5}$$

where x_t is the current input series or features, h_{t-1} is the last LSTM unit, f_t, is the forget gate, σ is the sigmoid function of the forget gate and W_f and b_f are the weight matrices and bias, respectively, of the forget gate.

In the second step, the sigmoid function is used in the input layer to decide what new information should be stored in the cell state and to update the cell state. Here, a tanh function is also used to create a vector of new candidate values, $\tilde{C}_t$, from the old cell state. Next, to update the new cell state, the values of sigmoid and tanh layers are combined. The equation for the input and tanh layers can be expressed by:

$$i_t = \sigma(W_i \cdot [h_{t-1}, x_t] + b_i) \tag{6}$$

$$\tilde{C}_t = tanh(W_C \cdot [h_{t-1}, x_t] + b_C) \tag{7}$$

$$C_t = f_t * C_{t-1} + i_t * \tilde{C}_t \tag{8}$$

where i_t is the input gate, σ is the sigmoid function of the input gate, W and b are the weight matrices and bias, respectively, of the cell state, and C_t is the new cell state.

Finally, the sigmoid layer decides what should be the output and which parts of the cell state are going to be output values. Then, cell state is added through tanh to get the values between -1 and 1 and multiply it by the output of the sigmoid gate. Here, the equation for the output gate and values can be expressed by:

$$o_t = \sigma(W_o \cdot [h_{t-1}, x_t] + b_o) \tag{9}$$

$$h_t = o_t * tanh(C_i) \tag{10}$$

where o_t is the output gate, W_o and b_o are the weight matrices and bias, respectively, of the output gate, σ is the sigmoid function of the output gate, and h_t is the output values.

3. Figures

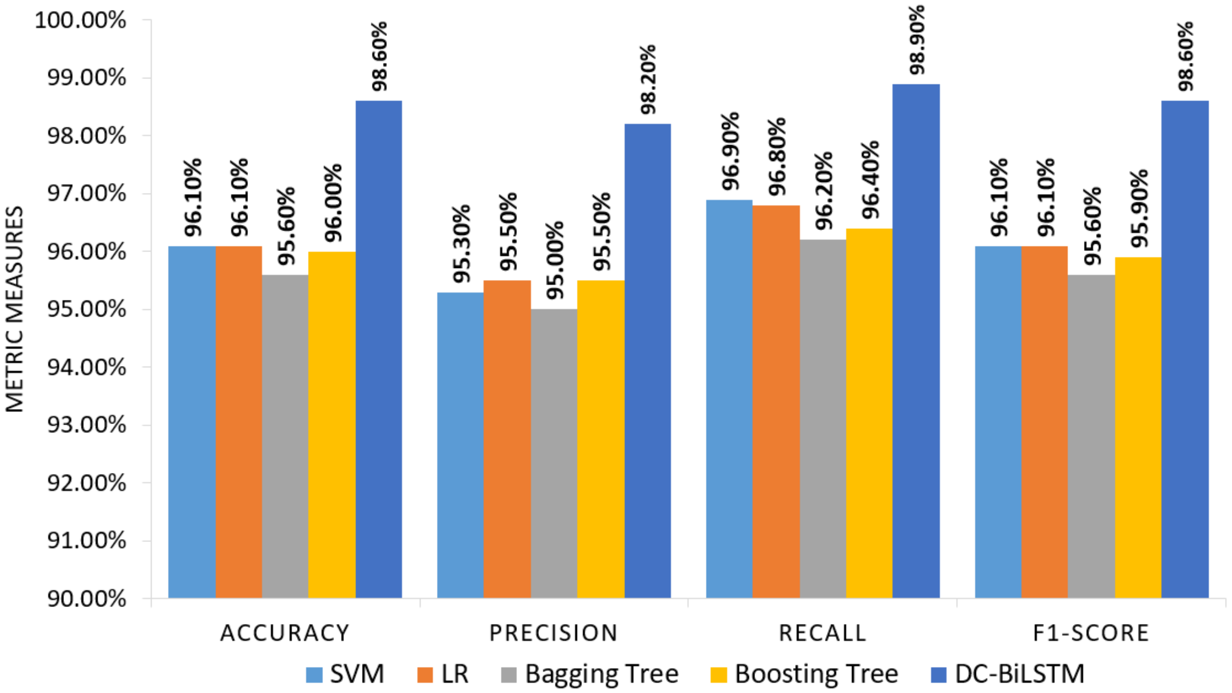

(a)

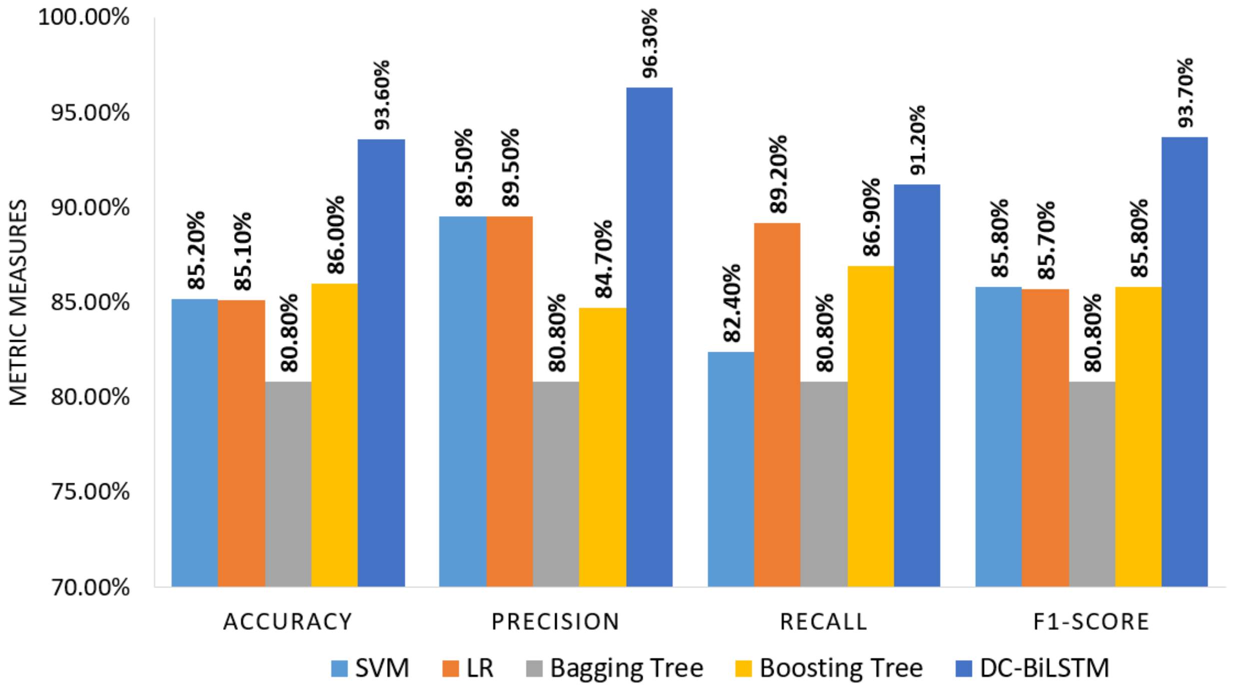

(b)

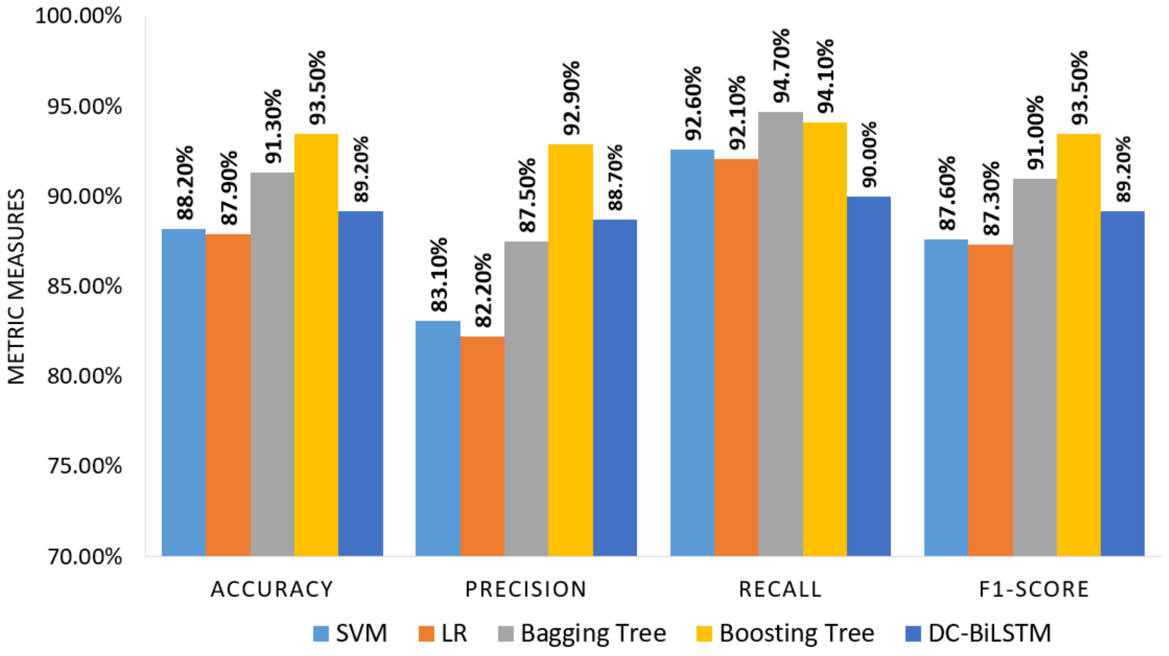

(c)

Figure S4. Comparative analysis graphs of four different evaluation metrics that show the results of binary classification obtained using different AI models. (**a**) The performance of internal test set (benign vs. malignant). (**b**) The performance of the internal test set (grade 3 vs. grade 5). (**c**) The performance of the external test set (benign vs. malignant). SVM: support vector machine, LR: logistic regression, and DC-BiLSTM: dual-channel bidirectional long short term memory.
